# LDL Receptor Knock-Out Mice Are a Physiological Model Particularly Vulnerable to Study the Onset of Inflammation in Non-Alcoholic Fatty Liver Disease

**DOI:** 10.1371/journal.pone.0030668

**Published:** 2012-01-25

**Authors:** Veerle Bieghs, Patrick J. Van Gorp, Kristiaan Wouters, Tim Hendrikx, Marion J. Gijbels, Marc van Bilsen, Jaap Bakker, Christoph J. Binder, Dieter Lütjohann, Bart Staels, Marten H. Hofker, Ronit Shiri-Sverdlov

**Affiliations:** 1 Department of Molecular Genetics, Pathology, Physiology and Clinical Genetics of Nutrition and Toxicology Research (NUTRIM) and Cardiovascular Research (CARIM) Institutes of Maastricht, University of Maastricht, Maastricht, The Netherlands; 2 Univ Lille Nord de France; Inserm U1011; UDSL; Institut Pasteur de Lille; Lille, France; 3 Center for Molecular Medicine, Austrian Academy of Sciences, and Department of Laboratory Medicine, Medical University of Vienna, Vienna, Austria; 4 Institute of Clinical Chemistry and Clinical Pharmacology, University of Bonn, Bonn, Germany; 5 Department of Pathology & Laboratory Medicine, University Medical Center Groningen, University of Groningen, Groningen, The Netherlands; Institute of Hepatology London, United Kingdom

## Abstract

**Background & Aims:**

Non-alcoholic steatohepatitis (NASH) involves steatosis combined with inflammation, which can progress into fibrosis and cirrhosis. Exploring the molecular mechanisms of NASH is highly dependent on the availability of animal models. Currently, the most commonly used animal models for NASH imitate particularly late stages of human disease. Thus, there is a need for an animal model that can be used for investigating the factors that potentiate the inflammatory response within NASH. We have previously shown that 7-day high-fat-high-cholesterol (HFC) feeding induces steatosis and inflammation in both *APOE2ki* and *Ldlr^−/−^* mice. However, it is not known whether the early inflammatory response observed in these mice will sustain over time and lead to liver damage. We hypothesized that the inflammatory response in both models is sufficient to induce liver damage over time.

**Methods:**

*APOE2ki* and *Ldlr^−/−^* mice were fed a chow or HFC diet for 3 months. C57Bl6/J mice were used as control.

**Results:**

Surprisingly, hepatic inflammation was abolished in *APOE2ki* mice, while it was sustained in *Ldlr^−/−^* mice. In addition, increased apoptosis and hepatic fibrosis was only demonstrated in *Ldlr^−/−^* mice. Finally, bone-marrow-derived-macrophages of *Ldlr^−/−^* mice showed an increased inflammatory response after oxidized LDL (oxLDL) loading compared to *APOE2ki* mice.

**Conclusion:**

*Ldlr^−/−^* mice, but not *APOE2ki* mice, developed sustained hepatic inflammation and liver damage upon long term HFC feeding due to increased sensitivity for oxLDL uptake. Therefore, the *Ldlr^−/−^* mice are a promising physiological model particularly vulnerable for investigating the onset of hepatic inflammation in non-alcoholic steatohepatitis.

## Introduction

Non-alcoholic fatty liver disease covers a disease spectrum ranging from simple steatosis to non-alcoholic steatohepatitis (NASH), liver fibrosis, cirrhosis and hepatocellular carcinoma [1]. Whereas steatosis might not adversely affect outcome, inflammation determines the long-term prognosis of this disease [2]–[5]. It is still not known why some patients progress towards inflammation, while others do not.

Exploring the molecular basis of the hepatic alterations associated with the metabolic syndrome is highly dependent on the availability of animal models which mimic the human condition from the physiological and metabolic points of view [6], [7]. To date, the most commonly used animal models for NASH imitate particularly late stages of human disease. Thus, there is a need for animal models that can be used for investigating the factors that potentiate the inflammatory response within NASH.

Non-alcoholic fatty liver disease (NAFLD) is a component of the metabolic syndrome and therefore it is frequently associated with hyperlipidemia and atherosclerosis [8]. One of the commonly used models for atherosclerosis studies is the low density lipoprotein (LDL) receptor knock-out (*Ldlr^−/−^)* mouse. The LDL receptor plays a major role in the clearance of apoB and apoE-containing lipoproteins [9]. Another mouse model for atherosclerosis is the apolipoprotein E2 knock-in (*APOE2ki*) mouse. In *APOE2ki* mice, the murine *apoe* gene is replaced by the human *APOE2* allele. The APOE2 protein has a markedly reduced affinity for the LDL receptor, leading to a plasma lipoprotein profile resembling human type III hyperlipoproteinaemia (HLP) [10]. Previously, we used these ‘humanized’ *APOE2ki* mice and *Ldlr^−/−^* mice to study NASH. Both hyperlipidemic mice developed early hepatic inflammation and steatosis when fed a high-fat-high-cholesterol (HFC) diet, whereas C57Bl6 mice only developed steatosis [11]. Unlike the lipoprotein profile in wild-type (WT) profile, in which most cholesterol is present in the HDL fraction, the profile of the *Ldlr^−/−^* mouse and *APOE2ki* is more comparable with the human plasma lipoprotein profile, in which cholesterol is mainly confined to the LDL fraction [12]. Thus, when fed a HFC diet, both *APOE2ki* and *Ldlr^−/−^* mice have the potential to be used as animal models for investigating the factors that potentiate the inflammatory response within NASH. However, data from the literature regarding the effect of inflammation on liver damage in hyperlipidemic mice on NASH progression are partial and inconclusive.

Based on the pronounced inflammatory response observed upon short-term HFC feeding, we hypothesized that the inflammatory response in both models will sustain over time and will be sufficient to induce fibrosis and liver damage. To test this hypothesis, male *APOE2ki* and *Ldlr^−/−^* mice were fed a HFC diet for 3 months and normolipidemic C57Bl6 (WT) mice were used as the control group. Surprisingly, although plasma and liver lipid levels were still elevated in both hyperlipidemic models after 3 months of HFC feeding, the inflammatory response was only sustained in the *Ldlr^−/−^* mice. In addition, increased apoptosis and hepatic fibrosis was only demonstrated in *Ldlr^−/−^* mice. By analysing the effect of oxidized LDL (oxLDL) loading on macrophages from the different models, we showed that these differences are most likely attributable to an increased sensitivity for oxLDL-induced inflammation in *Ldlr^−/−^* mice compared to *APOE2ki* mice. All together, the *Ldlr^−/−^* mice are a promising physiological model particularly vulnerable for investigating the onset of hepatic inflammation in the context of fatty liver disease.

## Materials and Methods

### Ethics statement

This study was carried out in strict accordance with the recommendations in the Guide for the Care and Use of Laboratory Animals of the National Institutes of Health. The protocol was approved by the Committee for Animal Welfare of Maastricht University (Permit Number: 2008-060). The investigation conforms to the Guide for the Care and Use of Laboratory Animals published by the US National Institutes of Health (NIH Publication No. 85–23, revised 1996).

### Mice and diet

The mice were housed under standard conditions and given free access to food and water. Twelve-week-old male C57Bl/6, *APOE2ki* and *Ldlr^−/−^* mice (with the same genetic background, C57Bl/6) were fed a high-fat-high-cholesterol diet (HFC) for 3 months (n = 8) containing 21% milk butter, 0.2% cholesterol, 46% carbohydrates and 17% casein. The control groups for each genotype were kept on a standard chow diet for the same period (3.3% fat, 46.1% carbohydrates and 19% protein). The 7 days and 3 month experiments were carried out at the same time. The collection of blood, sacrificing of the mice, and tissue isolation were performed as described previously [11].

### Lipid analysis

Liver and plasma lipid analysis were performed as described previously [11].

### Liver histology

Frozen liver sections (7 µm) were stained for infiltrated macrophages (macrophage marker, Mac-1), CD68 Kupffer cells (CD68 marker, FA11), T cells (T-cell marker, KT3), fibroblasts (fibroblast marker, ERTR7) and neutrophils (neutrophil marker, NIMP) as described previously [13]. TUNEL staining for apoptosis was performed on frozen liver sections according to the manufacturers' protocol (*In situ* Cell Death Detection Kit, Roche Applied Science). Paraffin-embedded liver sections (4 µm) were stained with Sirius Red as described previously [13] and α-smooth muscle actin (αSMA) (M0851, DAKO, Glostrup, Denmark). Horse-anti-mouse IgG (PI-2000, Vector Laboratories, Burlingame, USA) was used as secondary antibody and daminobenzidine (DAB) was applied as color substrate.

### Gene expression analysis

Total RNA isolation from mouse liver tissues, cDNA synthesis and Q-PCR analysis were performed as described previously [11].

### Measuring auto-antibody titers against modified LDL

Specific antibody titers against modified LDL in plasma were determined as described elsewhere [14], [15]. Plasma was diluted and antibody binding measured by chemiluminescent enzyme linked immunosorbent assay (ELISA).

### Measuring aminotranferases

The level of aminotransferases ALT in plasma of each individual mouse was measured by using the Reflotron-system and the test strips for ALT measurments (Roche Diagnostics, Almere, The Netherlands), according to the manufacturers instructions.

### Caspase 3/7 activity in liver homogenates

To detect apoptosis in liver homogenates, Caspase-Glo® 3/7 assay (#G8091, Promega, Madison, USA) was applied according to the manufacturers instructions. An equal volume of liver homogenate (5 µg/µl protein) was mixed with the Caspase-Glo® Reagent in 96-well, white-walled plates. The assay was incubated for 1.5 hour at room temperature before reading on a plate-reading luminometer.

### Enzyme-linked immunosorbent assay for TNF and IL6

TNF (0.05 µg/µl protein) and IL6 (0.1 µg/µl protein) were measured in liver homogenates by ELISA assays (CMC3013 and CMC0063 resp., Invitrogen, Camarillo, USA), according to the manufacturers instructions. Absorbance was measured at 450 nm using a microtiterplate reader (BioRad, Hercules, CA).

### Hydroxyproline assay

Hydroxyproline content of proteins was measured after acid hydrolysis with 6 M HCl for 5 hours. Samples were introduced into a tandem mass spectrometer using UPLC. Amino acids were measured in multiple reaction mode in ESI-positive mode. The mass transition 131.75>85.9 was used for the identification of hydroxyproline. Stable isotope-labelled asparagine was used as internal standard.

### *In vitro* murine macrophage culture

Bone marrow cells were isolated from the femurs and tibiae of C57Bl6, *APOE2ki* and *Ldlr^−/−^* mice. The cells were cultured in RPMI-1640 (GIBCO Invitrogen, Breda, the Netherlands) with 10% heat-inactivated fetal calf serum (Bodinco B.V., Alkmaar, the Netherlands), penicillin (100 U/ml), streptomycin (100 ug/ml), and L-glutamine (2 mM) (all GIBCO Invitrogen, Breda, the Netherlands) (R10) supplemented with 15% L929-conditioned medium (LCM) for 8–9 days to generate bone marrow-derived macrophages (BMM), as described previously [16]. The cells were treated for 24 h with 25 µg/ml of oxidized LDL (Intracel, Frederick, USA). Uptake of oxLDL particles that were DiI-labelled was assessed by flow cytometry after residual oxLDL was washed away.

### Statistical analysis

The data was statistically analysed by performing two-tailed non-paired *t*-tests using GraphPad Prism, version 4.03 for Windows. Data were expressed as the mean ±SEM and considered significant at p<0.05. *, ** and *** indicate p<0.05, 0.01 and 0.001, respectively.

## Results

### After 3 months of the HFC diet, hepatic inflammation is only sustained in *Ldlr^−/−^* mice

**Figure 1:**
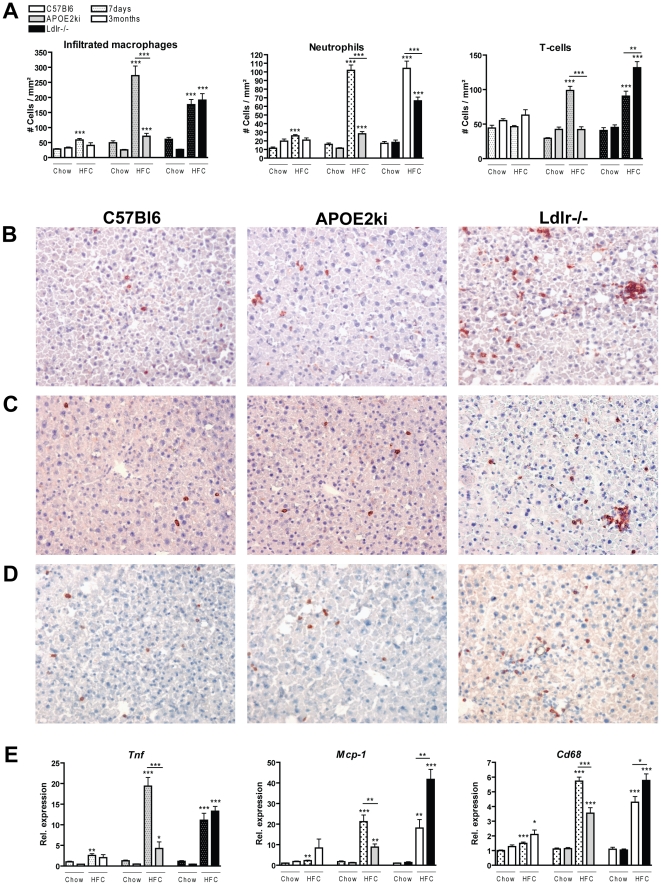
Parameters of hepatic inflammation. (A) Liver sections were stained for infiltrated macrophages and neutrophils (Mac-1), neutrophils (NIMP) and T cells (CD3) and counted as cells/mm^2^. (B–D) Representative pictures of Mac-1, NIMP and CD3 stained liver sections of control C57Bl6, *APOE2ki* and *Ldlr^−/−^* mice after 3 months of HFC diet, respectively (×200 magnification). (E) Hepatic gene expression of *tumor necrosis factor (Tnf), monocyte chemotactic protein 1 (Mcp-1)* and *Cd68*. Dotted bars indicate the earlier time point of 7 days. *, ** and *** indicate p<0.05, 0.01 and 0.001, respectively.

To determine whether hepatic inflammation was sustained in both *Ldlr^−/−^* and *APOE2ki* mice after 3 months (long-term) of HFC feeding, the numbers of infiltrated macrophages, neutrophils and T cells and the gene expression levels of *Tnf*, *Mcp-1* and *Cd68* in these mice were compared with control mice fed chow and with mice on the 7-day (short-term) HFC diet (Fig. 1). Normolipidemic C57Bl6 mice were used as the control group. To follow the changes in hepatic inflammation within time, we included the data of the 7-day HFC diet (dotted bars) in the first figure, which originates from Wouters *et al* [11]. These data indicated that both *Ldlr^−/−^* and *APOE2ki* mice had increased inflammation after 7 days of HFC feeding compared to mice on the chow diet and normolipidemic C57Bl6 mice, as reflected by elevated numbers of infiltrated macrophages, neutrophils and T cells. In control mice, only a minor increase in the number of infiltrated cells was observed after 7 days of HFC feeding (Fig. 1A). Gene expression analysis for *Tnf*, *Mcp-1* and *Cd68* confirmed these findings. Comparing 7 days and 3 months of HFC feeding demonstrated that the inflammatory response was only sustained in *Ldlr^−/−^* mice. In contrast, the number of infiltrated macrophages, neutrophils and T cells was significantly reduced in *APOE2ki* mice after 3 months of HFC feeding (Fig. 1A). Moreover, the inflammatory cells were more clustered in *Ldlr^−/−^* mice compared to *APOE2ki* and *C57Bl6* mice (Fig. 1B–D). In addition, the observed differences in hepatic inflammation between the hyperlipidemic models and between short- and long-term HFC diets were confirmed by gene expression analysis for *Tnf*, *Mcp-1* and *Cd68*, where the inflammatory response was sustained in the *Ldlr^−/−^* mice and abolished in the *APOE2ki* mice after 3 months of the HFC diet (Fig. 1E). Hepatic TNF and IL6 protein levels were also confirmed by ELISA (Fig. S1).

### Hepatic steatosis is present in all models after 3 months of the HFC diet

**Figure 2:**
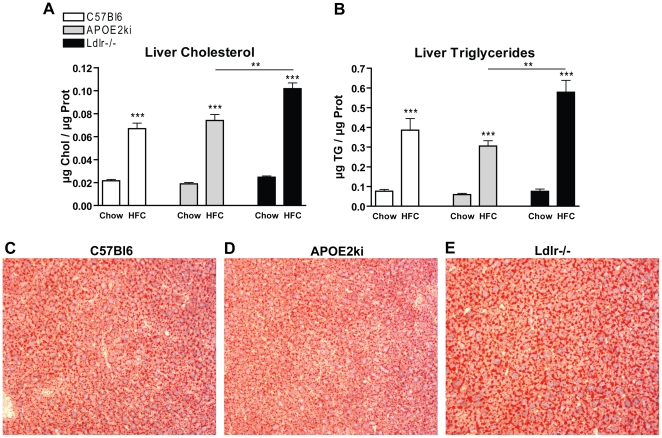
Parameters of hepatic steatotis. (A+B) Liver cholesterol and triglycerides after chow and 3 months of HFC diet in C57Bl6, *APOE2ki* and *Ldlr^−/−^* mice. (C–E) Representative pictures (×200 magnification) of the Oil red O staining after the chow diet and 3 months of the HFC diet in C57Bl6, *APOE2ki* and *Ldlr^−/−^* mice.

To investigate if the inflammatory response was associated with steatosis after 3 months of the HFC diet, liver lipid levels and Oil red O staining were performed on the livers of the three different mouse models. In general, HFC feeding induced weight gain in all models, but there were no significant differences between the 3 groups (Fig. S2). The liver cholesterol and triglyceride levels were increased in all *C57Bl6*, *APOE2ki* and *Ldlr^−/−^* mice after HFC feeding compared to mice on the chow diet. Furthermore, the levels of cholesterol and triglycerides were significantly higher in *Ldlr^−/−^* mice compared to *C57Bl6* and *APOE2ki* mice on HFC diet (Fig. 2A+B). These findings were confirmed by Oil red O staining (Fig. 2C–E).

### Only *Ldlr^−/−^* mice have increased fibrosis and apoptosis after 3 months of the HFC diet

**Figure 3:**
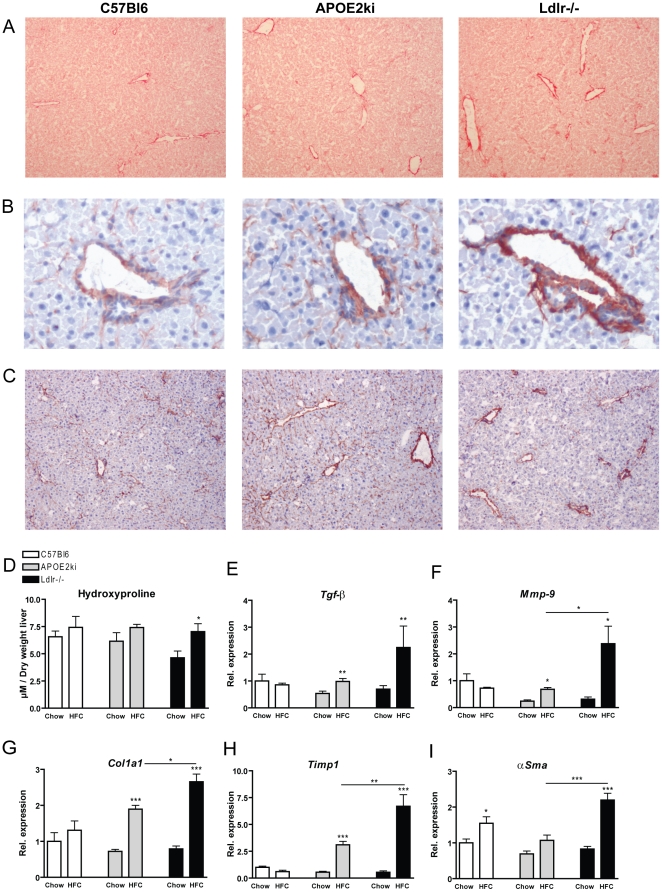
Parameters of hepatic fibrosis. (A–C) Representative pictures of (A) Sirius Red (magnification×100) and (B+C) ERTR7 (fibroblast marker) (magnification ×400, ×100, respectively) positive sections after 3 months on the HFC diet in C57Bl6, *APOE2ki* and *Ldlr^−/−^* mice, respectively. (D) Hepatic hydroxyproline content of C57Bl6, *APOE2ki* and *Ldlr^−/−^* mice after 3 months of chow and HFC diet. (E–I) Gene expression analysis of tumor growth factor beta *(Tgf-β)*, Collagen 1a1 *(Col1a1)*, metalloproteinase *(Mmp-9*), tissue inhibitor of metalloproteinase 1 (*Timp1*) and alpha smooth muscle actin (*αSMA*). Data were set relative to the C57Bl6 mice on the chow diet. * Significantly different from chow group. *, ** and *** indicate p<0.05, 0.01 and 0.001, respectively.

One of the features of advanced stages of NASH is hepatic fibrosis. To investigate whether the 3 mouse models differed in the extent of fibrosis, Sirius Red, fibroblast (ERTR7) and αSMA (activated hepatic stellate cells) staining were performed (Fig. 3A–C). The Sirius Red and fibroblast staining demonstrated that *Ldlr^−/−^* mice have increased hepatic fibrosis after 3 months on the HFC diet compared to C57Bl6 and *APOE2ki* mice. Likewise, the collagen content and fibroblast presence were greater around the portal areas in *Ldlr^−/−^* mice (Fig. 3C). However, in *APOE2ki* mice, ERTR7 staining is mainly prevalent in pericellular locations. This is probably also the reason why we couldn't detect a clear difference between the hydroxyproline levels of *APOE2ki* and *Ldlr^−/−^* mice (Fig. 3D). Nevertheless, the *Ldlr^−/−^* mice were the only model that demonstrated a significant difference between chow and HFC diet compared to C57Bl6 and *APOE2ki* mice. The αSMA staining was mainly prevalent around the portal areas in all 3 models and only showed activated stellate cells in a few, but not all, *Ldlr^−/−^* mice (Fig. S3). The histological findings were confirmed by the gene expression analysis of *Tgf-β*, *Mmp-9*, *Col1a1*, *Timp1* and *αSma* (Fig. 3E–I) and therefore we can conclude that the *Ldlr^−/−^* mice have more fibrosis compared to C57Bl6 and *APOE2ki* mice, although fibrosis is rather mild.

**Figure 4:**
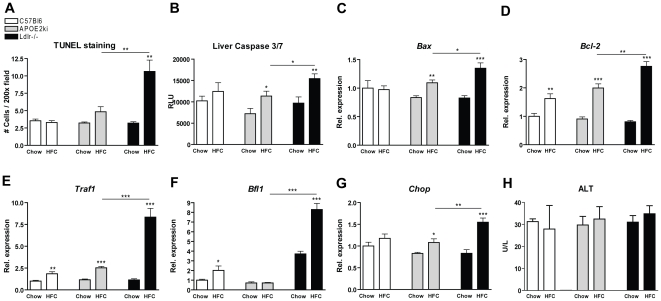
Parameters of apoptosis. (A) Scoring of TUNEL stained liver sections for apoptosis after chow and 3 months of HFC diet in C57Bl6, *APOE2ki* and *Ldlr^−/−^* mice. (B) Caspase 3/7 activity in liver homogenates. (C–G) Gene expression analysis of the apoptotic genes *Bax*, *Bcl-2*, *Traf1*, *Bfl1* and *Chop*. (H) Aminotransferase (ALT) levels in plasma. Data were set relative to the C57Bl6 mice on the chow diet. * Significantly different from chow group. *, ** and *** indicate p<0.05, 0.01 and 0.001, respectively.

Cell death by apoptosis is thought to give rise to larger regions of liver damage. To test whether hepatic inflammation was associated with apoptosis, liver sections were stained for apoptosis (TUNEL staining). After 3 months of HFC feeding, *Ldlr^−/−^* mice had increased apoptosis compared to *C57Bl6* and *APOE2ki* mice (Fig. 4A+Fig. S4). These findings were confirmed by increased levels of caspase 3/7 activity in the livers of *Ldlr^−/−^* mice compared to *APOE2ki* mice (Fig. 4B). Moreover, gene expression analysis of the apoptotic markers *Bax*, *Bcl-2*, *Traf1*, *Bfl1* and *Chop* confirmed these findings, indicating a clear association between the inflammatory response and apoptosis (Fig. 4C–G). However, the presence of elevated transaminases in plasma like alanine aminotransferase (ALT) did not differ between the different models (Fig. 4H).

### High plasma cholesterol levels and foamy Kupffer cells in both hyperlipidemic models after 3 months of the HFC diet

Plasma cholesterol levels were significantly increased after 3 months of HFC feeding in all three models. No differences were observed between *APOE2ki* and *Ldlr^−/^* ^−^ mice (Fig. S5A). Moreover, the size of foamy KCs did not differ between the hyperlipidemic models. The KCs of these models were swollen compared to chow and *C57Bl6* mice on HFC diet (Fig. S5B).

### *Ldlr^−/−^* mice have increased hepatic expression levels of lipid-related genes

**Figure 5:**
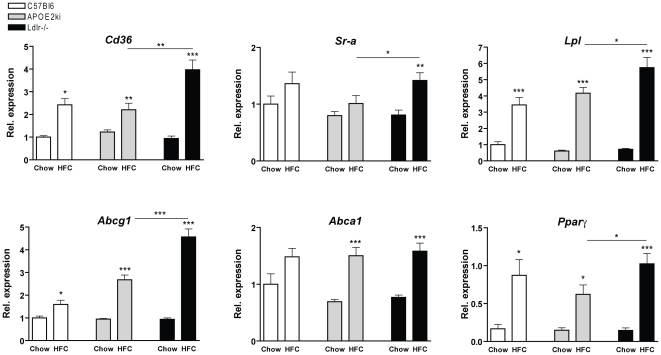
Cholesterol-related hepatic gene expression. Gene expression analysis of *Cd36*, scavenger receptor A *(Sr-a)*, lipoprotein lipase *(Lpl)*, ATP binding cassette G1 *(Abcg1)* and A1 *(Abca1)* and peroxisome proliferator activated receptor *(Ppar-γ)* after chow and 3 months of HFC diet in C57Bl6, *APOE2ki* and *Ldlr^−/−^* mice. Data were set relative to the C57Bl6 mice on the chow diet. * Significantly different from chow group. *, ** and *** indicate p<0.05, 0.01 and 0.001, respectively.

To further investigate the effect of HFC feeding on hepatic cholesterol metabolism, gene expression levels of the lipid-related genes were analysed. As expected, the hepatic expression of *Cd36*, *Sr-a*, *Lpl*, *Abcg1*, *Abca1* and *Pparγ* were all elevated in mice upon HFC diet. In addition, *Ldlr^−/−^* mice on the HFC diet showed higher expression levels of these genes (except for *Abca1*) than *APOE2ki* mice (Fig. 5).

### Bone marrow-derived macrophages of *Ldlr^−/−^* mice are more inflammatory after oxidized LDL loading compared to *APOE2ki* macrophages

**Figure 6:**
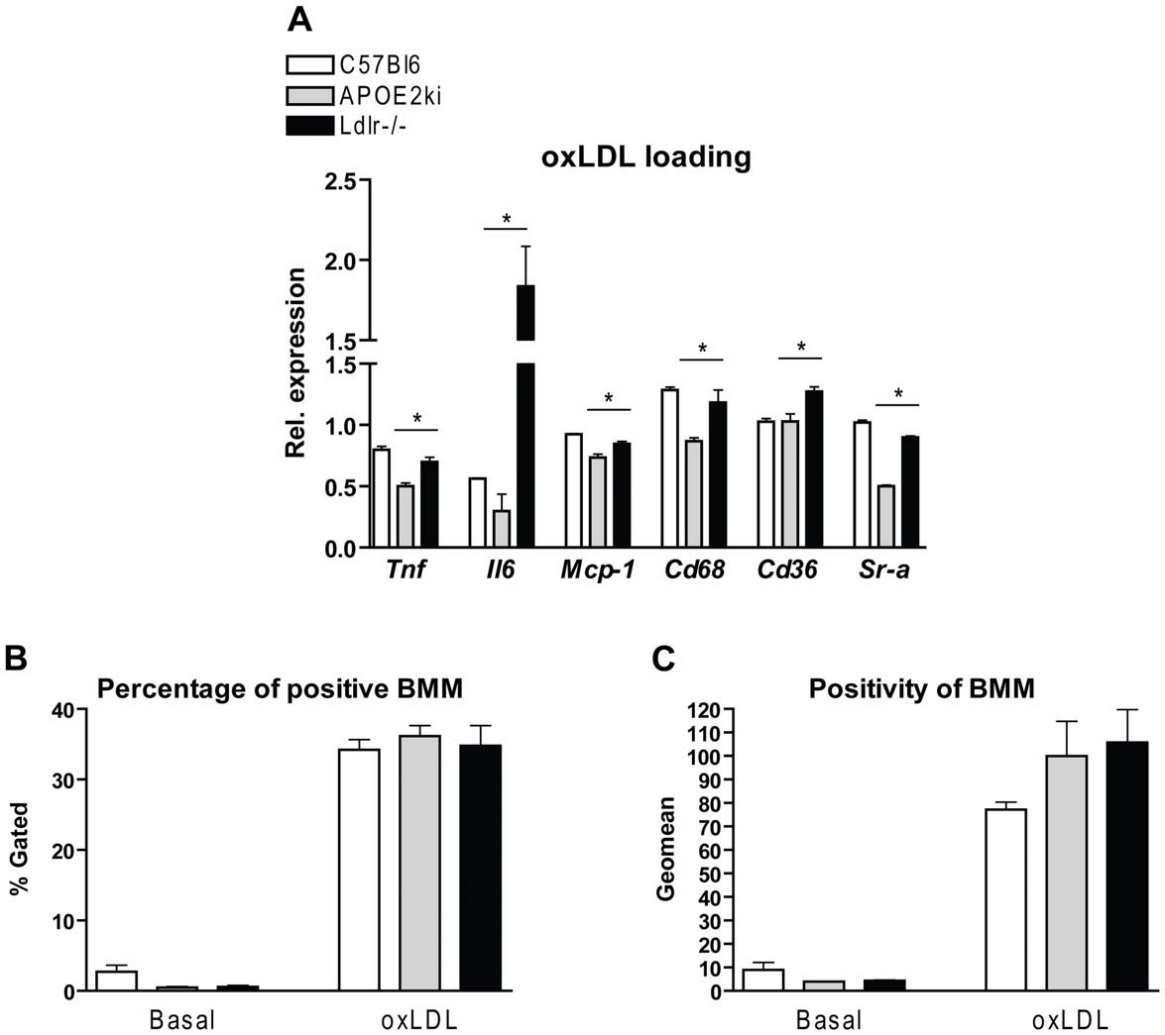
Loading of bone marrow-derived macrophages with oxLDL. (A) Bone marrow-derived macrophages (BMM) of C57Bl6, *APOE2ki* and *Ldlr^−/−^* mice were loaded with oxidized low-density lipoprotein (oxLDL) and gene expression of tumor necrosis factor *(Tnf)*, interleukin 6 *(Il6)*, monocyte chemotactic protein 1 *(Mcp-1)*, *Cd68*, *Cd36* and scavenger receptor A *(Sr-a)* was analysed. Data were set relative to the basal levels of C57Bl6 bone marrow. (B) Percentage of positive BMM at basal levels and after oxLDL loading. (C) Positivity of BMM at basal levels and after oxLDL loading. * indicate p<0.05.

We have previously shown that the level of naturally occurring antibodies against oxLDL in the plasma correlates with hepatic inflammation [13]. To investigate whether the differences in hepatic inflammation between *Ldlr^−/−^* and *APOE2ki* correlate with the plasma levels of these antibodies, measurements of IgM and IgG antibodies against oxLDL were performed. These levels were comparable between the three different mouse models, suggesting a similar generation of oxidation-specific epitopes (Fig. S6A+B). Moreover, bone marrow-derived macrophages (BMM) of these mice were loaded with oxLDL, which leads to foam cell formation and inflammation *in vitro*. Gene expression analysis of the inflammatory gene *Tnf*, *Il6*, *Mcp-1* and *Cd68* after oxLDL loading demonstrated that *APOE2ki* BMM have a lower expression compared to *C57Bl6* and *Ldlr^−/−^* BMM. Additionally, the two main scavenger receptors responsible for the uptake of modified lipids, *Cd36* and *Sr-a*, showed increased expression in *Ldlr^−/−^* compared to *APOE2ki* (Fig. 6A). The percentage of positive BMM and the positivity of BMM were increased upon oxLDL loading compared to basal levels, but were not different between the groups (Fig. 6B–C). In conclusion, although the uptake of oxLDL was the same between the different models, BMM of *APOE2ki* mice were less inflammatory compared to *C57Bl6* and *Ldlr^−/−^* mice.

## Discussion

The present study illustrates that the *Ldlr^−/−^* mice are a promising long term NASH model, while in contrast to our expectations, *APOE2ki* mice are not. The HFC diet led to sustained hepatic inflammation, apoptosis and fibrosis in *Ldlr^−/−^* mice, but not in *APOE2ki* mice. This difference was most likely a consequence of the increased sensitivity for oxLDL-induced inflammation in *Ldlr^−/−^* mice compared to *APOE2ki* mice. These novel observations indicate that hyperlipidemia and steatosis are not sufficient for maintaining the inflammatory response in the liver. In addition, our data demonstrate that the *Ldlr^−/−^* mouse model can be used as an excellent physiological model particularly vulnerable for investigating hepatic inflammation in the context of fatty liver disease.

Lack of a suitable animal model that faithfully recapitulates the pathophysiology of human NASH is a major obstacle in delineating mechanisms responsible for the progression of steatosis to NASH. The best characterized and most widely used genetic model for NASH is the leptin-deficient mouse (ob/ob). The ob/ob mice develop hepatic steatosis but not hepatic inflammation or fibrosis, possibly due to the loss of normal leptin signalling [17]. Therefore, these ob/ob mice need a pro-injurious stimulus, such as an endotoxin (LPS) [18]. The most well-known nutritional model for NASH is a diet deficient in methionine and choline (MCD). These mouse models display all of the hallmarks of NASH, from steatosis to inflammation and fibrosis development [19]. However, mice fed an MCD-deficient diet tend to lose weight and display lowered plasma TG levels and are therefore very different from NASH in human metabolic syndrome or diabetes patients who are mostly obese and/or hyperlipidemic [6]. Therefore, the currently available genetic and nutritional models are especially useful to investigate late stages of NASH but have some limitations restricting their use to serve as a physiological model to study the development of hepatic inflammation in the context of NASH [6],[8].

Based on the analogy between the mechanisms of NASH and atherosclerosis, an emerging trend in NASH research is to utilize the mouse models traditionally targeted for studies of atherosclerosis, including *APOE2ki* and *Ldlr^−/−^* mice. The *APOE2ki* mouse, a humanized mouse model of hyperlipidemia, has outstanding potential as it is highly responsive to dietary factors and pharmacological interventions [20]. As these mice are not commonly used, the effect of feeding a long-term HFC diet on the liver of *APOE2ki* mice had so far not been investigated. In contrast, several studies have been performed to determine the consequences of a long-term HFC diet on *Ldlr^−/−^* mice. The *Ldlr^−/−^* mice are mildly hypercholesterolemic due to the absence of LDL receptors, which prolongs the plasma half-life of VLDL and LDL [21]. These mice were utilized by Kong *et al.*, who revealed that 5 months of HFC feeding in male *Ldlr^−/−^* mice induced macrovesicular steatosis, but not inflammatory cell infiltration [22]. In contrast, Yoshimatsu *et al.* demonstrated that 3 months of HFC feeding of female *Ldlr^−/−^* mice resulted in steatosis, infiltration of neutrophils into the liver and increased serum aminotransferases (ALT) levels. However, the diet was highly enriched with cholesterol (1.25%) and cholic acid (0.5%), and the latter is a primary bile acid that is known to cause hepatic toxicity. In addition, the effect on hepatic fibrosis was not investigated [23]. Finally, Gupte *et al.* demonstrated that middle-aged (12-month-old) male *Ldlr^−/−^* mice fed an HFC diet for 3 months developed steatosis, inflammation, fibrosis, oxidative stress and elevated liver injury markers [24]. However, the authors concluded that the advanced age of these mice exacerbated the HFC-induced fibrosis [25]. Thus, the current data regarding the effect of a long-term HFC diet on NASH progression in hyperlipidemic mice is partial and inconclusive. Besides, elevated liver enzymes such as ALT in the blood usually suggest liver damage, however, there is not always a correlation between these liver enzymes and NAFLD or NASH [26]. By using young mice with a physiological diet, we proved for the first time that the *Ldlr^−/−^* mice are a promising model for investigating the onset of hepatic inflammation in NASH and can be a valuable tool for conducting interventional studies; these findings should eventually lead to a better understanding of human NASH and the development of an efficient therapy for this condition.

Studies in *apoE^−/−^* mice show that ApoE influences several inflammatory processes due to the fact that it is produced by a wide variety of cell types, including macrophages [27]–[30]. However, our data suggest that the APOE2 isoform is not directly involved in inflammation as the inflammatory response was not sustained in the *APOE2ki* mice. Unlike *APOE2ki* mice, *apoE^−/−^* mice have been extensively used as models for atherosclerosis. It was shown that the mean cross-sectional area of the plaque in the *APOE2ki* mice is approximately half that seen in age-matched *apoE^−/−^* mice with a similar genetic background and the plaques of the *APOE2ki* mice are less mature [31]–[33]. Moreover, it was also demonstrated that apoE has allele-specific effects in protecting cells from oxidative cell death, with E2 the most effective one [34]. In addition, the circulation time of atherogenic particles is also reduced in *APOE2ki* mice compared to *apoE^−/−^* mice , which can result in an decreased inflammatory response in the body [35], [36]. Altogether, these evidences suggest that *APOE2ki* mice are less inflammatory compared to the *apoE^−/−^* mice.

Where *Ldlr^−/−^* mice require an atherogenic diet to develop atherosclerosis, *APOE2ki* mice spontaneously develop the full spectrum of atherosclerotic lesions, even on a regular chow diet. Moreover, an atherogenic diet, high in fat and cholesterol, further exacerbates the development of atherosclerosis and xanthomas in these *APOE2ki* mice. However, it was shown that the lesions in these *APOE2ki* mice mainly consisted of foam cells and had relatively fewer fibrous caps, cholesterol clefts and necrotic cores [10]. Thus, in line with our observations, it is likely that the foamy macrophages from the atherosclerotic plaques of APOE2ki mice are less inflammatory compared to those in *Ldlr^−/−^* mice. Currently, *APOE2ki* mice are still accepted as an established model for atherosclerosis. Altogether, based on our data and data from the literature, this paradigm should be re-evaluated.

Although hepatic inflammation was completely abolished in *APOE2ki* mice after 3 months of HFC feeding, plasma and liver lipid levels were still elevated. Previously, we reported that elevated plasma cholesterol levels can trigger hepatic inflammation [11]. In addition, omitting cholesterol from the diet even resulted in a dramatic inhibition of hepatic inflammation, without affecting the levels of steatosis. In line with these findings about steatosis, recent reports have also raised doubts about steatosis as a precondition for the development of inflammation during NASH progression [37], [38] [39]. In the present study, neither plasma cholesterol, steatosis, nor anti-oxLDL antibodies were correlated with hepatic inflammation. These observations suggest that the differences in inflammation are not related to systemic difference in lipids and oxidation, but rather to differences in the activity of intracellular inflammatory pathways. In line with this hypothesis, KCs from both hyperlipidemic models still had a foamy appearance after 3 months of HFC diet. Moreover, BMM of *Ldlr^−/−^* mice showed increased expression of *Il-6* and *Cd36* compared to macrophages from *APOE2ki* and *C57Bl6* mice after oxidized LDL loading. Relevantly, IL-6 was shown to dictate the transition from acute to chronic inflammation by changing the nature of leukocyte infiltration (from polymorphonuclear neutrophils to monocyte/macrophages). In addition, IL-6 exerts stimulatory effects on T- and B-cells, thus favoring chronic inflammatory responses [40]. Thus, these data suggest that, unlike macrophages from *APOE2ki* and *C57Bl6* mice, macrophages of *Ldlr^−/−^* mice produce higher levels of IL-6 and therefore are more prone to develop chronic inflammation. Relevantly, IL6 is also increased in serum of patients with NAFLD and is linked with insulin resistance [41]. Gene expression of *Cd36*, the main scavenger receptor responsible for the uptake of oxidized cholesterol and involved in inflammatory signal transduction, was also higher in BMM of *Ldlr^−/−^* mice after oxLDL loading compared to APOE2ki and *C57Bl6* mice. Interestingly, oxLDL up-regulates CD36 expression via the peroxisome proliferator-activated receptor (PPAR) γ, and this may initiate a feed forward loop of CD36 expression that amplifies the inflammatory response [42]. In support of this view, the expression levels of several target genes for PPARγ (ABCG1, LPL, CD36) were also reduced in the livers of APOE2ki mice compared to *Ldlr^−/−^* mice. Thus, it is possible that this loop mechanism does not occur in APOE2ki mice and therefore these mice are less sensitive to the inflammatory cascade.

So far, there is no suitable physiological model for studying the hepatic inflammation in a metabolic context that faithfully recapitulates the pathophysiology of human NASH. In this study, the hepatic inflammatory response induced by prolonged HFC feeding in *APOE2ki* and *Ldlr^−/−^* mice was investigated. We demonstrated that *Ldlr^−/−^* mice have increased sensitivity for oxLDL-induced inflammation, apoptosis and fibrosis compared to *APOE2ki* mice. Therefore, the *Ldlr^−/−^* mouse model is particularly useful for understanding the relationships between lipid metabolism and inflammatory recruitment in the context of NASH. This model may therefore be an excellent platform for the assessment of therapeutic strategies for hepatic inflammation.

## Supporting Information

Figure S1 **TNF and IL6 ELISA.** Hepatic protein levels of TNF and IL6 in liver homogenates of C57Bl6, *APOE2ki* and *Ldlr^−/−^* mice. * Significantly different from chow group. ** and *** indicate p<0.01 and 0.001, respectively.
(TIF)

Figure S2 **Weight.** Relative weight gain after 3 months of HFC diet in C57Bl6, *APOE2ki* and *Ldlr^−/−^* mice.
(TIF)

Figure S3 **Staining for activated hepatic stellate cells.** The αSMA staining for activated hepatic stellate cells in C57Bl6, *APOE2ki* and *Ldlr^−/−^* mice after 3 months of HFC feeding.
(TIF)

Figure S4 **Apoptosis.** Representative pictures (200× magnification) of TUNEL stained liver sections of C57Bl6, *APOE2ki* and *Ldlr^−/−^* mice after 3 months of HFC feeding.
(TIF)

Figure S5 **Plasma cholesterol levels and foamy Kupffer cells.** (A) Plasma total cholesterol levels after chow and 3 months of the HFC diet in the three different models. (B) Representative pictures (magnification ×200) after 3 months of the HFC diet for C67Bl6, *APOE2ki* and *Ldlr^−/−^* mice, respectively. * Significantly different from chow group. * and *** indicate p<0.05 and 0.001, respectively.
(TIF)

Figure S6 **Antibodies against oxidized LDL in plasma.** (A) IgM auto-antibody titers to MDA-LDL and CuOx-LDL. (B) IgG auto-antibody titers to MDA-LDL and CuOx-LDL. * Significantly different from chow group. *, ** and *** indicate p<0.05, 0.01 and 0.001, respectively.
(TIF)
