# Supplementary figures and images for: LDL Receptor Knock-Out Mice Are a Physiological Model Particularly Vulnerable to Study the Onset of Inflammation in Non-Alcoholic Fatty Liver Disease
Source: PLoS One. 2012 Jan 25;7(1):e30668. doi: 10.1371/journal.pone.0030668 (PMC3266276; doi:10.1371/journal.pone.0030668)

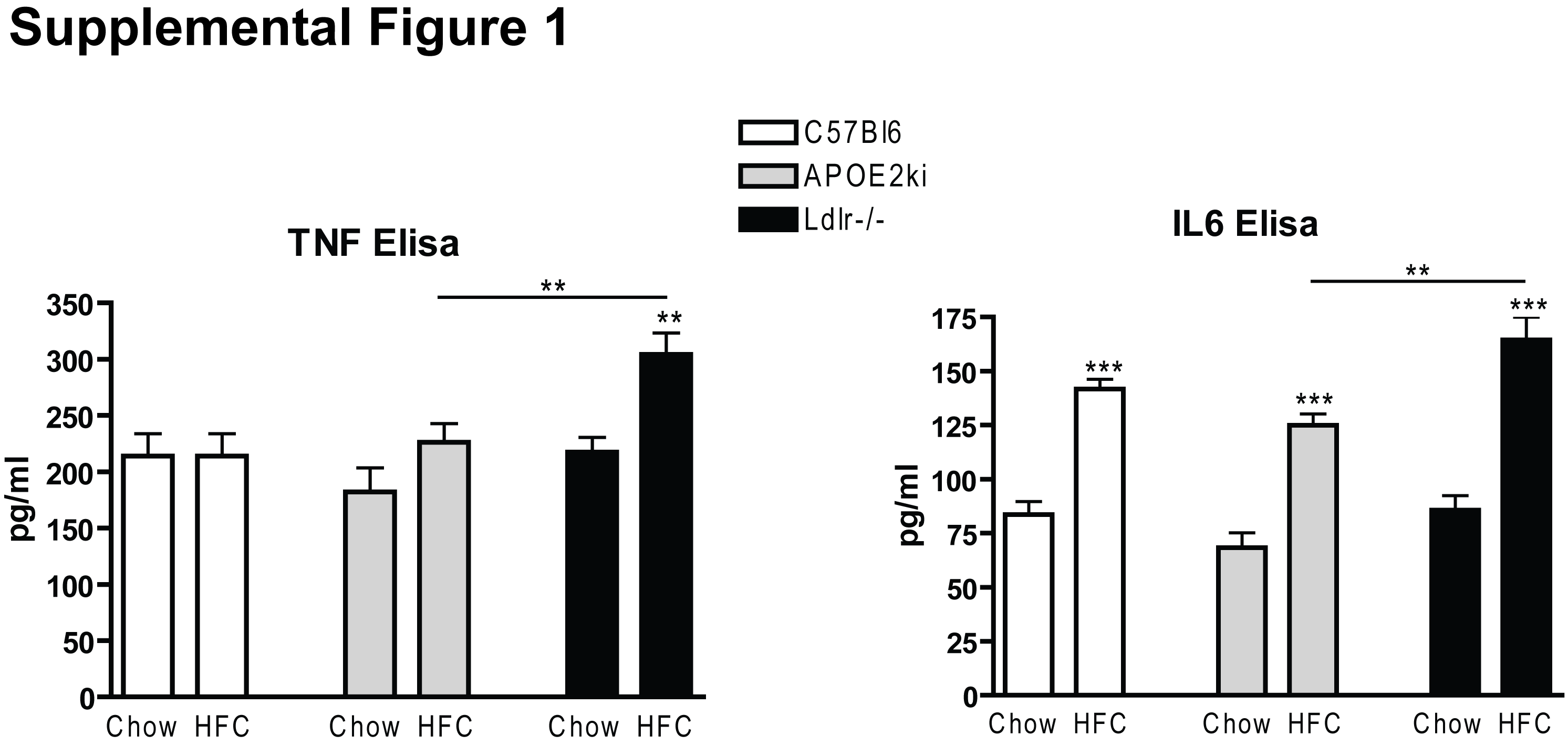

Supplement: Figure S1 — TNF and IL6 ELISA. Hepatic protein levels of TNF and IL6 in liver homogenates of C57Bl6, APOE2ki and Ldlr−/− mice. * Significantly different from chow group. ** and *** indicate p<0.01 and 0.001, respectively. (TIF) [file pone.0030668.s001.tif]

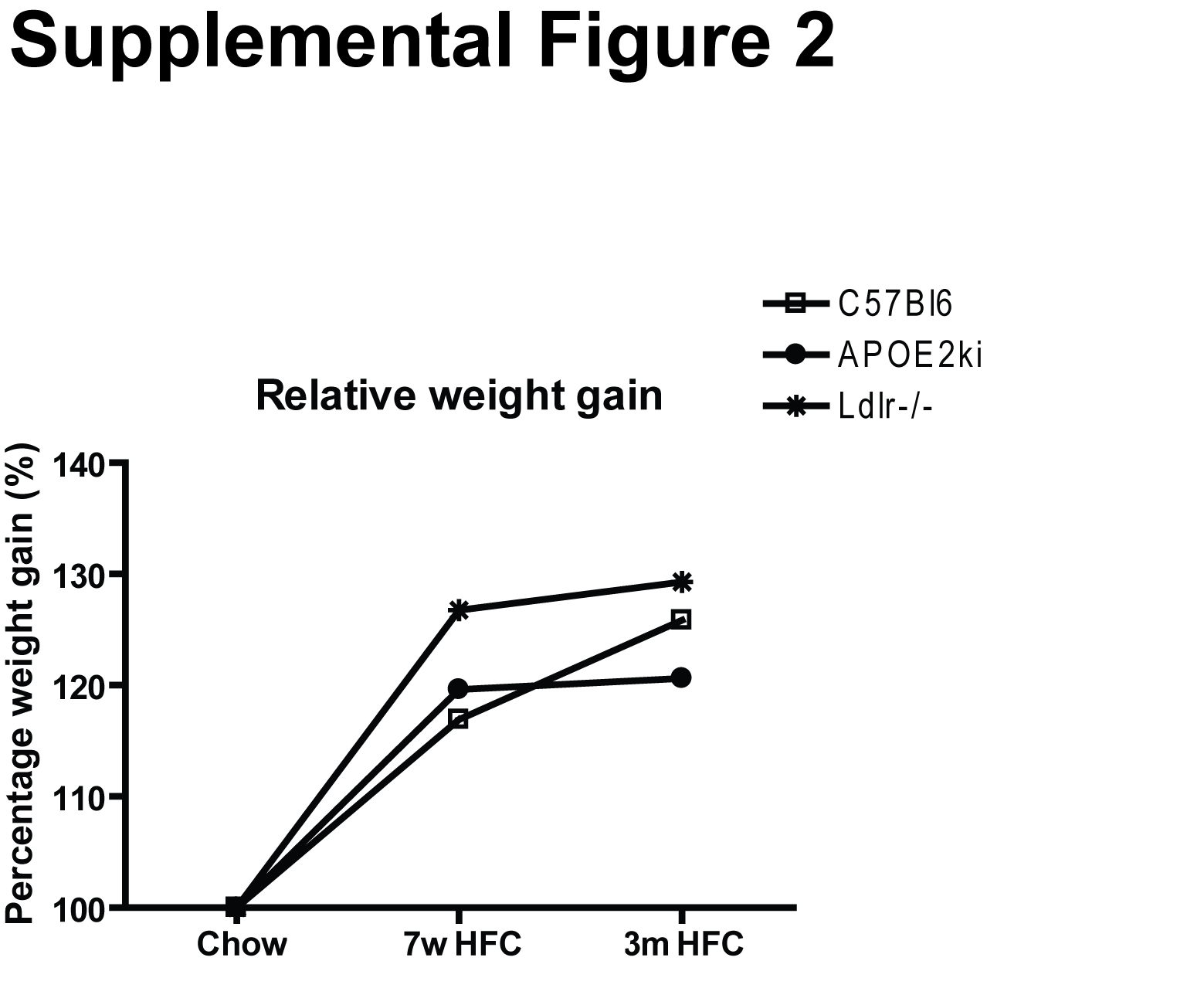

Supplement: Figure S2 — Weight. Relative weight gain after 3 months of HFC diet in C57Bl6, APOE2ki and Ldlr−/− mice. (TIF) [file pone.0030668.s002.tif]

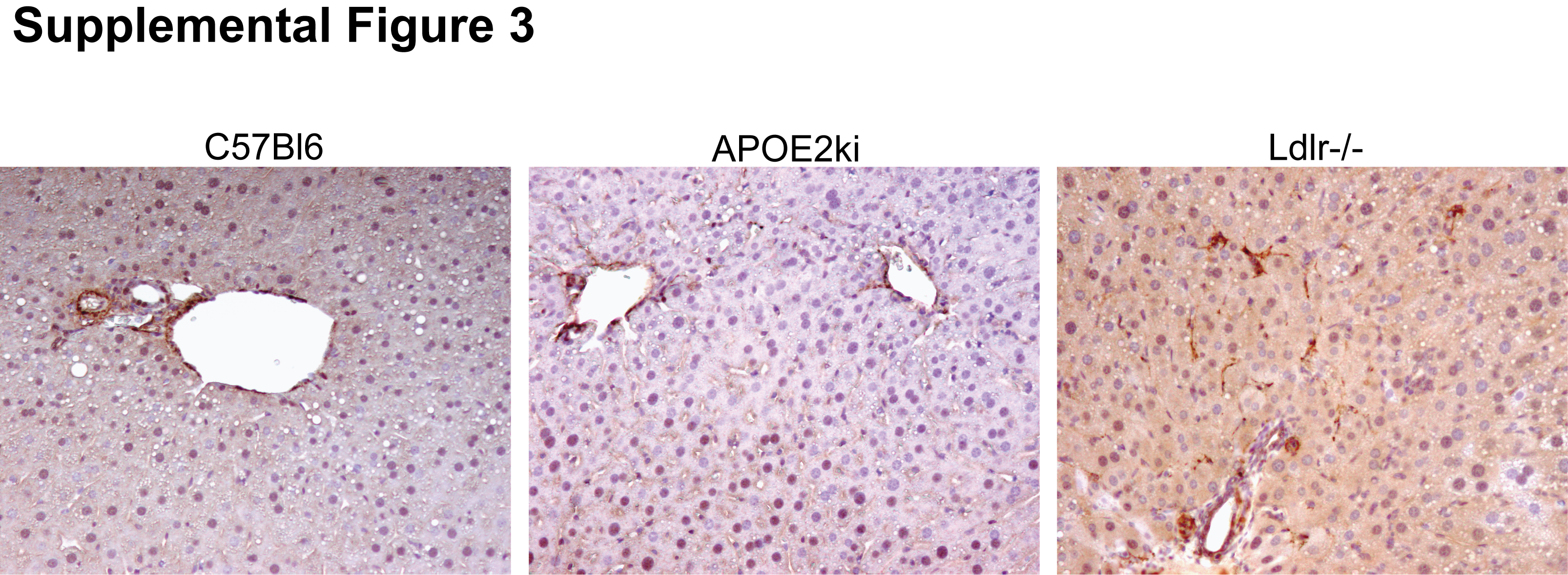

Supplement: Figure S3 — Staining for activated hepatic stellate cells. The αSMA staining for activated hepatic stellate cells in C57Bl6, APOE2ki and Ldlr−/− mice after 3 months of HFC feeding. (TIF) [file pone.0030668.s003.tif]

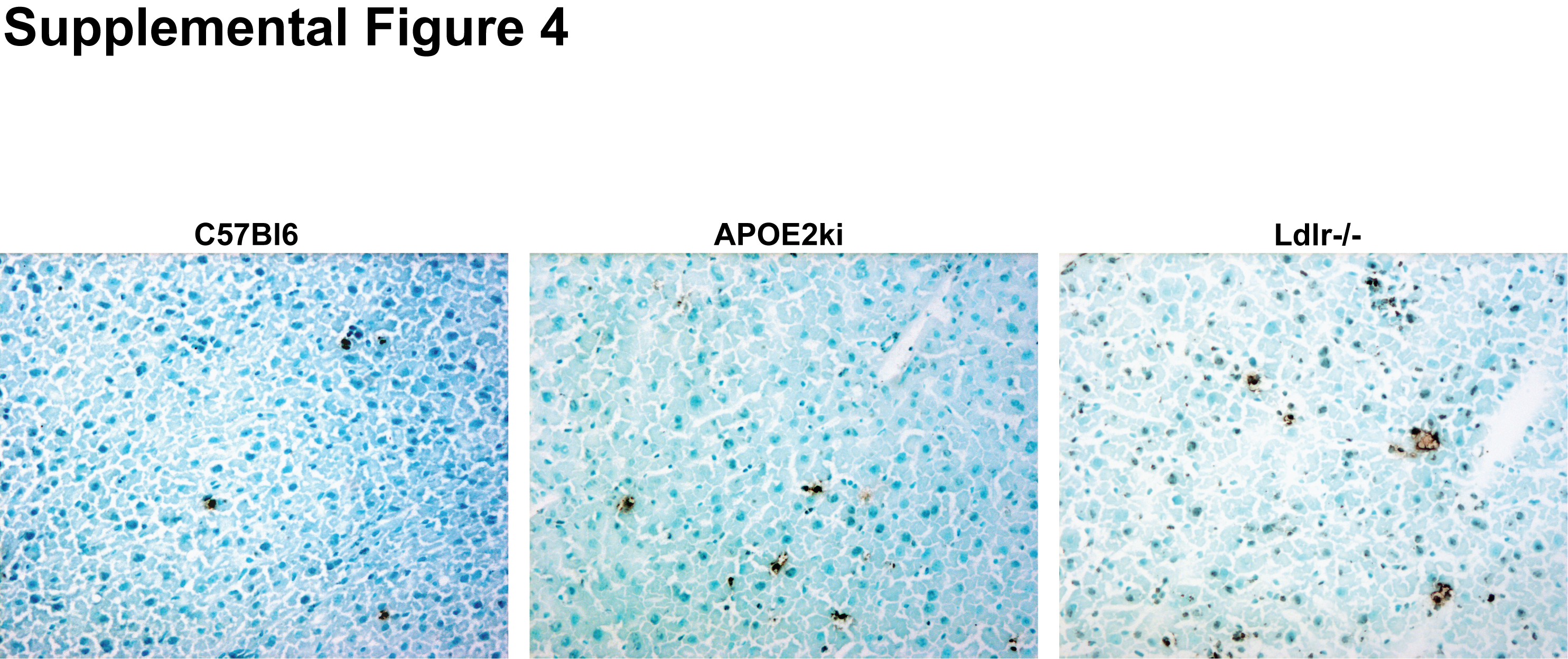

Supplement: Figure S4 — Apoptosis. Representative pictures (200× magnification) of TUNEL stained liver sections of C57Bl6, APOE2ki and Ldlr−/− mice after 3 months of HFC feeding. (TIF) [file pone.0030668.s004.tif]

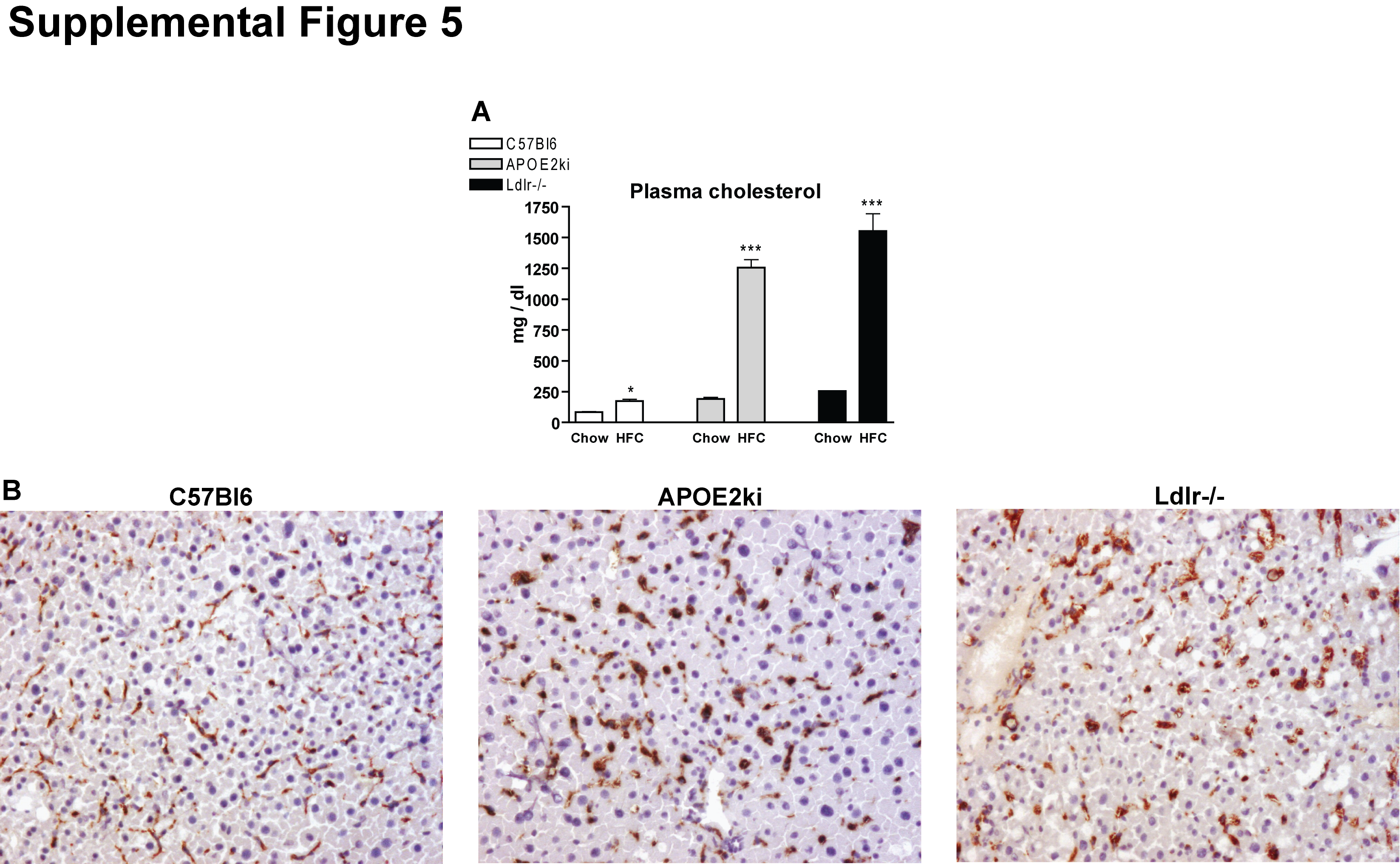

Supplement: Figure S5 — Plasma cholesterol levels and foamy Kupffer cells. (A) Plasma total cholesterol levels after chow and 3 months of the HFC diet in the three different models. (B) Representative pictures (magnification ×200) after 3 months of the HFC diet for C67Bl6, APOE2ki and Ldlr−/− mice, respectively. * Significantly different from chow group. * and *** indicate p<0.05 and 0.001, respectively. (TIF) [file pone.0030668.s005.tif]

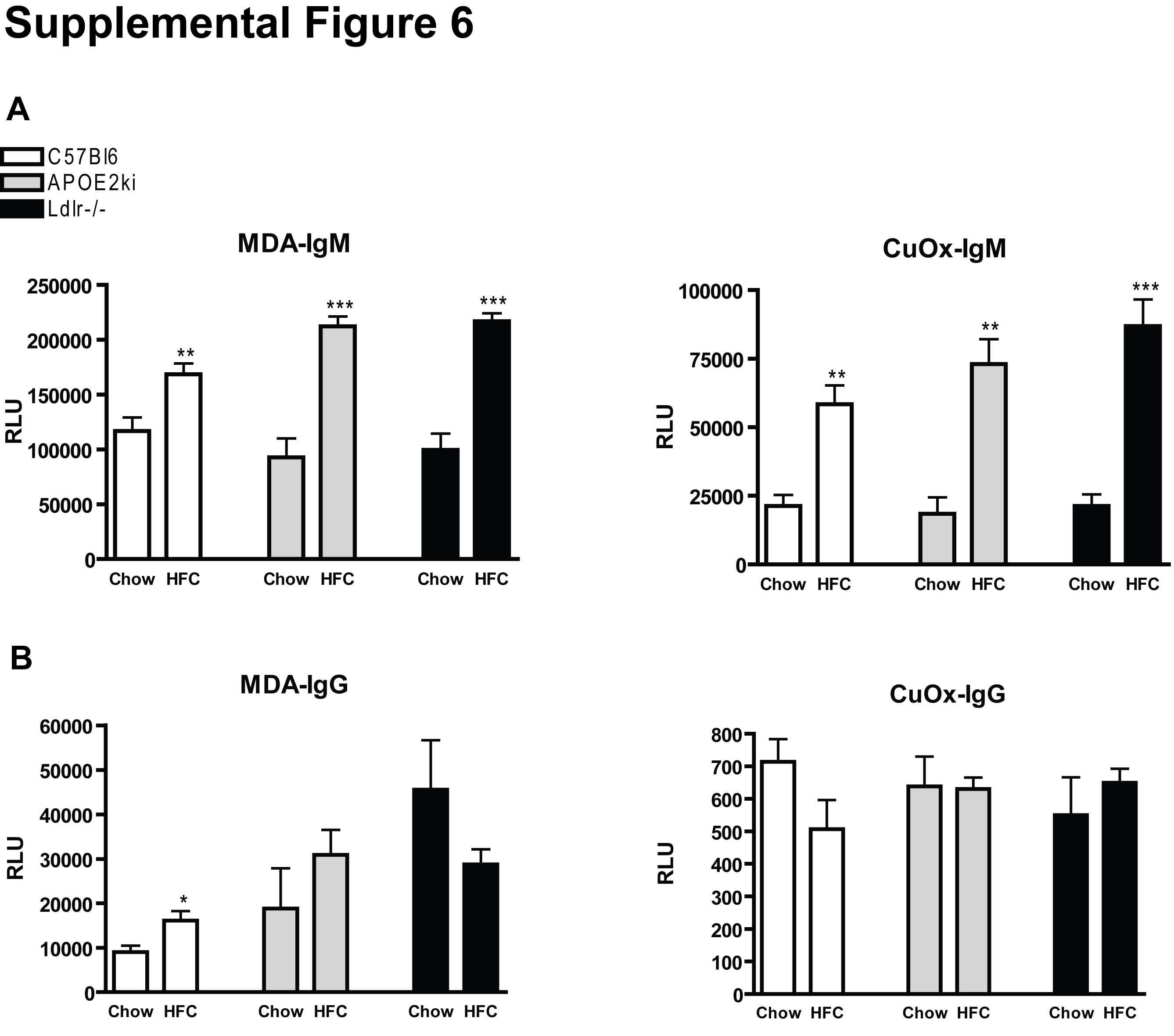

Supplement: Figure S6 — Antibodies against oxidized LDL in plasma. (A) IgM auto-antibody titers to MDA-LDL and CuOx-LDL. (B) IgG auto-antibody titers to MDA-LDL and CuOx-LDL. * Significantly different from chow group. *, ** and *** indicate p<0.05, 0.01 and 0.001, respectively. (TIF) [file pone.0030668.s006.tif]
